# ATP-Binding Cassette and Solute Carrier Transporters: Understanding Their Mechanisms and Drug Modulation Through Structural and Modeling Approaches

**DOI:** 10.3390/ph17121602

**Published:** 2024-11-27

**Authors:** Ahmad Elbahnsi, Balint Dudas, Isabelle Callebaut, Alexandre Hinzpeter, Maria A. Miteva

**Affiliations:** 1 Inserm U1268 MCTR, CiTCoM UMR 8038 CNRS, Université Paris Cité, 75006 Paris, France; 2 Muséum National d’Histoire Naturelle, UMR CNRS 7590, Institut de Minéralogie, de Physique des Matériaux et de Cosmochimie—IMPMC, Sorbonne Université, 75005 Paris, France; 3 CNRS, INSERM, Institut Necker Enfants Malades—INEM, Université Paris Cité, 75015 Paris, France

**Keywords:** SLC transporters, ABC transporters, cryo-electron microscopy, artificial intelligence modeling, molecular docking, molecular dynamics

## Abstract

The ATP-binding cassette (ABC) and solute carrier (SLC) transporters play pivotal roles in cellular transport mechanisms, influencing a wide range of physiological processes and impacting various medical conditions. Recent advancements in structural biology and computational modeling have provided significant insights into their function and regulation. This review provides an overview of the current knowledge of human ABC and SLC transporters, emphasizing their structural and functional relationships, transport mechanisms, and the contribution of computational approaches to their understanding. Current challenges and promising future research and methodological directions are also discussed.

## 1. Introduction

Membrane transporters play a crucial role in mediating communication between cells and their external environment. The ATP-binding cassette (ABC) and solute carrier (SLC) transporters represent two major superfamilies of these membrane transporters, each with distinct functions and mechanisms [1,2]. Both ABC and SLC transporters play pivotal roles in drug development, impacting numerous aspects of pharmacokinetics and pharmacodynamics. These transporters are integral in determining drug absorption, distribution, metabolism, and excretion (ADME) [3,4,5,6], which are key factors in the efficacy and safety profile of therapeutic agents.

ABC transporters are known for their ability to transport a wide range of substances across cell membranes, primarily functioning as exporters in eukaryotic cells. This efflux capability plays a significant role in drug disposition and resistance. For instance, the overexpression of certain ABC exporters in cancer cells can lead to multidrug resistance (MDR), a major challenge in chemotherapy. Understanding the interaction between drugs and these transporters is crucial for overcoming resistance and improving drug delivery to target tissues [7,8].

Conversely, SLC transporters mainly facilitate the influx or import of molecules into eukaryotic cells and are crucial for the uptake of nutrients and many drugs [3]. In prokaryotes, however, SLC transporters can operate in both directions depending on cellular needs. Targeting these transporters can enhance drug absorption and treatment specificity. For example, designing drugs that can be selectively taken up by certain SLC transporters in targeted tissues can lead to more effective treatments with reduced side effects.

In addition to their role in drug pharmacokinetics, both ABC and SLC transporters are involved in various physiological processes and pathological conditions. For example, mutations in ABC transporters can lead to diseases such as cystic fibrosis and Tangier disease [9,10], while SLC transporter dysfunctions are linked to conditions like diabetes and neurological disorders [11,12]. Therefore, a deep understanding of these transporters is essential for the advancement of personalized medicine and the development of next-generation drugs.

The prediction and development of inhibitors or modulators of these transporters present a promising avenue in drug development. Such agents can be used to increase drug bioavailability, overcome resistance mechanisms, or reduce toxicity by altering drug distribution. However, targeting these transporters also poses challenges, including the risk of drug–drug interactions (DDIs) and off-target effects [13]. Consequently, a comprehensive exploration of interactions with ABC and SLC transporters is now an integral part of the drug development process.

Both experimental and computational studies have significantly advanced our understanding of these transporters by revealing detailed insights into their structure, conformational dynamics, substrate specificity, potential binding sites, and interactions with small-molecule modulators and substrates. Structural biology techniques, such as cryo-electron microscopy (cryo-EM) and X-ray crystallography, have provided detailed insights into the transporter mechanisms [1,14,15]. Meanwhile, computational approaches, including molecular dynamic (MD) simulations and docking studies, have offered dynamic views and predictive models that complement experimental findings [4,16,17].

This review presents the current knowledge of human ABC and SLC transporters, focusing on their key biological roles and implications in medical conditions. It explores the specific structure–function relationships of ABC and SLC transporters, including their transport mechanisms, drug pharmacokinetics, and resistance. Detailed insights are provided into CFTR (ABCC7), an anion channel implicated in cystic fibrosis, and FPN1 (ferroportin 1/SLC40A1), the sole known iron exporter in humans, highlighting their unique roles as ion transporters within ABC and SLC transporter superfamilies. By integrating structural knowledge from both experimental and computational perspectives, this review offers a holistic view of the roles of ABC and SLC transporters in drug development and therapeutic applications. Finally, key findings, current challenges, and promising future research and methodological directions are discussed.

## 2. Membrane Transporters: Key Biological Roles and Implication in Medical Conditions

### 2.1. ABC Transporters

The ABC transporter superfamily comprises highly prevalent proteins distributed across various cellular tissues, where they facilitate the translocation of a wide array of endogenous and exogenous compounds [1,18]. These transported substances encompass hormones, vitamins, lipids, peptides, ions, xenobiotics, and drugs. ABC transporters operate by harnessing the energy derived from the binding and hydrolysis of adenosine triphosphate (ATP) molecules, allowing them to transport these compounds against their concentration gradients [19,20]. Dysfunctions or excessive expression of ABC transporters have implications in various medical conditions, which make them important targets in drug discovery [21,22].

Members of the ABCB, ABCC, and ABCG subfamilies are particularly significant due to their roles in drug and xenobiotic transport, mediating the unidirectional efflux of drugs. This property is crucial in conferring MDR to cancer cells [7], as well as in regulating drug pharmacokinetics at critical physiological barriers, such as the blood–brain barrier, liver, kidney, and intestine [8,21]. Inhibiting these transporters can lead to drug–drug interactions (DDIs) and impact drug efficiency and safety [23]. Some of these transporters are also explored as therapeutic targets for various conditions, including cystic fibrosis or intrahepatic cholestasis [9,24].

In the B subfamily, ABCB1—multidrug resistance 1 (MDR1) or P-glycoprotein (P-gp)—is a polyspecific multidrug transporter crucial for cellular detoxification [18,25]. Cancer cells can exploit the functions of ABCB1 to develop MDR during chemotherapy, significantly reducing treatment effectiveness [26,27]. As the first identified ABC efflux transporter in drug-resistant tumors, ABCB1 is associated with resistance to a broad range of anticancer drugs. Efforts to inhibit ABCB1 have evolved through three generations of compounds. First-generation inhibitors like verapamil displayed high toxicity at effective doses [28]. Second-generation inhibitors showed improved efficacy, but interacted with cytochrome P450 3A4, affecting co-administered drug profiles [29]. Third-generation inhibitors like tariquidar were designed to be effective at lower doses without being substrates for cytochrome P450 3A4 [30,31]. However the clinical failure of these inhibitors underscored the ongoing challenge of accurately predicting drug behavior in humans and highlights the necessity for more reliable predictive models for clinical outcomes.

ABCB4 (MDR3), primarily expressed in liver cells, shares 75% sequence identity with ABCB1 and transports phosphatidylcholine (PC) to bile canaliculi [32]. This process is essential for the formation of mixed micelles with co-secreted bile salts and cholesterol, mitigating the detergent activity of bile acids and preventing cholesterol gallstone formation, which can lead to inflammation and cholestasis [33]. Mutations in the ABCB4 gene are associated with conditions such as progressive familial intrahepatic cholestasis type 3 (PFIC3) and intrahepatic cholestasis of pregnancy [34,35,36]. Over 400 distinct disease-causing ABCB4 variants have been identified, presenting a challenge in finding pharmacological treatments for the severe forms of these diseases [37].

ABCB11, the bile salt export pump (BSEP), shares ~50% sequence identity with ABCB1, and is predominantly expressed in the canalicular membrane of hepatocytes. It is the principal transporter of bile salts [38]. Mutations or adverse drug reactions affecting ABCB11 can result in diseases such as progressive familial intrahepatic cholestasis type 2 (PFIC2) and drug-induced cholestasis (DIC) [39,40]. Severe PFIC2 often leads to early-onset cirrhosis, with a risk of hepatocellular carcinoma and cholangiocarcinoma before the age of one [41]. More than 300 clinical mutations have been identified in the ABCB11 gene from cholestatic patients (as recorded in the Human Gene Mutation database: http://www.hgmd.cf.ac.uk/, accessed on 20 October 2024).

Although there are currently no established treatments, certain mutations in ABCB4 and ABCB11 exhibit sensitivity to some pharmacological molecules (such as ABCC7/CFTR potentiators, as discussed below in this section), suggesting potential avenues for therapeutic intervention in these transporter-related cholestatic diseases. Despite their high sequence identity, ABCB1, ABCB4, and ABCB11 exhibit distinct substrate specificities, underscoring the importance of detailed structural studies to elucidate the precise molecular mechanisms underlying their transport activities.

In the C subfamily, ABCC1—multidrug resistance protein 1 (MRP1)—plays a pivotal role in the efflux of a variety of substrates, including drugs and their metabolites [42]. Similarly to P-gp, MRP1 contributes to MDR in cancer cells [43,44,45]. Additionally, it is involved in the transport of inflammatory mediators and has a significant role in the physiological response to oxidative stress [43]. The quest for inhibitors of MRP1 and other MRPs is crucial due to their significant involvement in MDR. Unlike P-gp, the knowledge about MRP1 inhibitors remains limited, marking a significant gap in cancer research. Nevertheless, studies have pursued various strategies to identify MRP1 inhibitors, including the exploration of phytochemical derivatives, miRNA-based therapies, and tyrosine kinase inhibitors (TKIs), as well as other small molecules. Phytochemicals like curcumin and flavonoids like genistein have demonstrated potential in inhibiting MRP1 activity. These substances are suggested to act by impacting the ATPase activity of MRP1, highlighting the therapeutic promise of natural compounds in combating drug resistance in cancer treatments [46,47].

Other important MRPs include ABCC2 (MRP2), crucial for biliary excretion of organic anions and associated with Dubin–Johnson syndrome [48]; ABCC3 (MRP3), which transports bile acids and conjugated bilirubin from the liver to the blood [49]; ABCC4 (MRP4) and ABCC5 (MRP5), which mediate the efflux of cyclic nucleotides and antiviral drugs [50]; and ABCC6 (MRP6), associated with pseudoxanthoma elasticum [51].

ABCC7 (CFTR) is unique within the ABC family, as it functions as an anion channel responsible for chloride and bicarbonate transport, essential for maintaining fluid homeostasis [9,52]. Mutations in the *CFTR* gene cause cystic fibrosis, a severe genetic disorder characterized by impaired mucociliary clearance and progressive respiratory failure [53,54]. Over 2000 CF mutations have been identified and categorized into six classes, each characterized by a specific type of defect: I—nonsense, II—folding, III—gating, IV—conductance, V—reduced synthesis, and VI—decreased stability [55,56]. Some mutations lead to combinatorial defects in CFTR channel biology, with implications for pharmacotherapy [55]. Hence, the most common mutation, F508del, leads to inefficient folding and impaired function, resulting in degradation of CFTR and subsequent dysregulation of epithelial fluid transport [57,58,59]. The discovery of small molecules targeting specific classes of mutations has been pivotal and delineated the druggable nature of mutated ABC transporters. Treatments like correctors (e.g., tezacaftor/VX-661 and elexacaftor/VX-445) targeting folding mutations and potentiators (e.g., ivacaftor/VX-770) targeting gating mutations have shown promise in restoring CFTR function [60,61,62,63,64,65,66,67]. The combination therapy Trikafta (elexacaftor/tezacaftor/ivacaftor) has demonstrated substantial improvements in clinical outcomes for patients with the F508del mutation [66,68,69,70,71]. However, challenges remain, particularly for mutations that are not fully responsive to current therapies [72]. Novel co-potentiators, including natural compounds, offer a potential avenue for improving the effectiveness of treatment in cases where current options are inadequate [73,74]. In addition to correctors and potentiators, CFTR inhibitors like CFTRinh-172 have been studied for their potential to regulate CFTR activity, particularly useful in conditions where CFTR function needs to be downregulated (such as secretory diarrhea caused by cholera toxins, where CFTR hyperactivation leads to excessive fluid and chloride secretion) [75]. They are also used in research to study CFTR’s role in various biological processes and to simulate CF features across different biological models [75].

Potentiators of CFTR, like ivacaftor/VX-770 or SBC molecules, have been proven to rescue other ABC transporters such as ABCB4 and ABCB11 [76,77,78,79,80], expanding the potential therapeutic impact of these compounds beyond CF.

In the G subfamily, ABCG2/BCRP (breast cancer resistance protein) is a significant multidrug exporter and gatekeeper at the blood–brain barrier, affecting the pharmacokinetics of various drugs [81,82,83]. Like P-gp, BCRP contributes to MDR in several cancer types [84,85]. It protects tissues by exporting numerous endogenous substrates and a broad variety of xenobiotics to extracellular spaces, such as the blood lumen at the blood–brain barrier [18]. BCRP inhibitors, which include a wide variety of chemicals with diverse structures, demonstrate significant therapeutic potential. Common inhibitors, such as elacridar, target both ABCB1/P-gp and BCRP effectively. In addition, HIV protease inhibitors and tyrosine kinase inhibitors (TKIs) like ritonavir and imatinib, respectively, exhibit dual inhibitory effects on these transporters. Furthermore, specific BCRP inhibitors like fumitremorgin C (FTC) and its derivatives demonstrate high selectivity and reduced toxicity, reflecting significant progress in targeting BCRP at the molecular level [82].

ABCG5 and ABCG8 form heterodimers that facilitate cholesterol transport from hepatocytes to bile canaliculi [86]. Defects in these transporters are linked to sitosterolemia, a recessive disorder characterized by elevated plasma sterol levels, increased sterol absorption, and reduced sterol excretion in bile [87,88].

Another important member of the G subfamily is ABCG1, which primarily regulates cholesterol homeostasis and has been implicated in neurological diseases such as Alzheimer’s, where it influences amyloid-beta levels and neuroinflammation [89].

### 2.2. SLC Transporters

SLC transporters play a pivotal role in transporting a wide range of substances, including nutrients, neurotransmitters, ions, drugs, and more across the plasma membrane. These transporters are crucial for various biological processes, such as the regulation of cell signaling and homeostasis [2,90]. SLC transporters are also significant in pharmacology, particularly in drug ADME in organs like the kidney and liver and at the blood–brain barrier [4,90]. Many SLCs operate as secondary active transporters, utilizing electrochemical gradients to transport substrates [90]. Recently, cryo-EM structures of the Na^+^/H^+^ exchanger, the SLC9C1 transporter essential for sperm motility and fertility, unveiled the unique solute carrier controlled by regulatory domains commonly found in voltage-gated ion channels [91,92]. Genetic mutations in SLCs have been linked to various diseases, including neurological disorders, metabolic conditions, and cancer [93]. Consequently, several SLC transporters are under investigation as potential drug targets, highlighting their growing importance in pharmaceutical research and development.

Despite their significance, SLCs remain underexplored due to the vast size and diversity of the SLC family. Many transporters still have unknown substrates, and only a few have well-characterized chemical modulators. Additionally, disease-associated mutations in SLCs are not well understood, complicating efforts to develop targeted therapies. However, recent EU-funded projects, such as RESOLUTE [94] (https://www.resolute-project.eu/) and EubOPEN (https://www.eubopen.org/), are aiming to address these gaps by systematically characterizing SLC functions and identifying novel substrates and modulators. These initiatives are expected to significantly advance our understanding of SLC biology and their potential as therapeutic targets [6,95].

SLC transporters significantly influence drug pharmacokinetics by mediating the ADME of various pharmaceutical agents. Key families of SLC transporters include the SLC22, SLC47, SLCO, SLC15, SLC29, SLC10, and SLC19 subfamilies, which play significant roles in drug pharmacokinetics [2,6,14,96,97].

The SLC22 family includes organic cation transporters (OCTs) and organic anion transporters (OATs), which are crucial for the renal and hepatic handling of drugs [98,99]. OCT1 (SLC22A1) and OCT2 (SLC22A2) facilitate the uptake of cationic drugs into the liver and kidneys, respectively. OCT1, primarily expressed in the liver, is crucial for hepatic uptake of cationic drugs, such as metformin. OCT2, mainly found in the kidneys, mediates the uptake of cationic drugs from the bloodstream into renal tubular cells, a crucial step for their subsequent excretion in urine [100]. OAT1 (SLC22A6) and OAT3 (SLC22A8) are essential for the renal excretion of a wide range of anionic drugs and endogenous compounds. Expressed in the renal proximal tubules, they facilitate the uptake of organic anions from the blood into renal cells, enabling their secretion into the urine. This function is vital for clearing drugs such as antiviral agents and diuretics from the body [98,99].

The SLC47 family, including multidrug and toxin extrusion proteins (MATEs), plays a crucial role in drug excretion [101,102,103]. MATE1 (SLC47A1) and MATE2-K (SLC47A2) are involved in the renal secretion of drugs and toxins into the urine by exchanging intracellular cationic drugs with extracellular protons [104,105]. MATE1 also contributes to the biliary excretion of drugs in the liver.

The SLCO subfamily, which includes organic anion transporting polypeptides (OATPs), also plays a significant role in drug pharmacokinetics [106,107]. OATP1B1 (SLCO1B1) and OATP1B3 (SLCO1B3) are predominantly expressed in the liver and mediate the hepatic uptake of a wide range of endogenous compounds and drugs, including statins and certain anticancer drugs. Genetic polymorphisms in OATP1B1/B3 can lead to altered drug pharmacokinetics and an increased risk of adverse drug reactions, underscoring the importance of these transporters in personalized medicine [108].

The SLC15 family includes peptide transporters (PEPTs), which are crucial for the absorption of peptide-like drugs [109,110,111,112]. PEPT1 (SLC15A1), predominantly expressed in the small intestine, mediates the absorption of di- and tripeptides, as well as peptide-like drugs such as beta-lactam antibiotics and angiotensin-converting enzyme inhibitors [111]. PEPT2 (SLC15A2) is mainly found in the kidneys, where it reabsorbs filtered peptides and peptide-like drugs from the tubular lumen, playing a key role in their renal handling [111].

The SLC29 family includes equilibrative nucleoside transporters (ENTs), which are involved in the uptake of nucleoside-derived drugs [113,114,115]. ENT1 (SLC29A1) [116] and ENT2 (SLC29A2) are expressed in various tissues (liver, kidneys, and intestines), where they facilitate the bidirectional transport of nucleosides and nucleoside analogue drugs across cellular membranes, contributing to their ADME [115].

The SLC10 family includes transporters that influence the enterohepatic recirculation of bile acids and impact the solubility and absorption of some drugs [117]. Human Na^+^–taurocholate co-transporting polypeptide (NTCP/SLC10A1), primarily expressed in the liver, mediates the uptake of bile acids from the blood into hepatocytes and serves as a cellular entry receptor for human hepatitis B and D viruses (HBV/HDV), making it a significant target for antiviral drug development [118,119]. ASBT (SLC10A2), expressed in the intestines, reabsorbs bile acids from the intestinal lumen, playing a critical role in maintaining bile acid homeostasis and influencing the pharmacokinetics of drugs undergoing enterohepatic circulation [120,121,122].

The SLC19 family is involved in the uptake of folate and antifolate drugs [123,124,125,126]. The reduced folate carrier (RFC, SLC19A1) is essential for the cellular uptake of folate and antifolate drugs. RFC is particularly important for the transport of methotrexate, a key antifolate drug used in cancer therapy [125].

While the aforementioned SLC transporters contribute to drug pharmacokinetics, many other SLC transporters are essential for diverse physiological processes unrelated to drug metabolism and disposition. Glucose transporters (GLUTs), belonging to the SLC2 family, are essential for glucose homeostasis, mediating the transport of glucose across cell membranes [15,127]. Ferroportin (FPN1/SLC40A1), a member of the SLC40 family, is the only known iron exporter in mammals and is essential for maintaining systemic and cellular iron homeostasis [128]. It is expressed in key iron-transporting cells, including macrophages, duodenal enterocytes, hepatocytes, and placental syncytiotrophoblasts [129]. FPN1 is regulated by the liver-derived peptide hepcidin, which triggers FPN1 internalization and degradation, thereby reducing iron release into the bloodstream. The hepcidin–ferroportin interaction is central to many inherited and acquired iron metabolism disorders [130,131,132]. Mutations in FPN1 cause hemochromatosis type 4, an autosomal dominant hereditary iron overload disorder [133]. Disease-causing mutations fall into two main categories: gain-of-function mutations, which impair hepcidin binding, and loss-of-function mutations, which affect iron export [130,134,135,136]. Additionally, specific FPN1 inhibitors can prevent iron overload by blocking FPN1 activity, and a recent structure was characterized in the presence of vamifeport, providing insights into its inhibitory mechanism and therapeutic potential [137,138].

## 3. Structure–Function Relationships in Membrane Transporters Based on Structural and Modeling Studies

Early 3D structure models of human ABC transporters were based on bacterial ABC transporters such as Sav1866 (a multidrug ABC transporter from *Staphylococcus aureus*) or MsbA (an ATPase in Gram-negative bacteria, initially identified as a multicopy suppressor of the *htrB* gene involved in lipid A biosynthesis) providing a foundation for understanding their basic transport mechanisms [139,140]. These bacterial templates facilitated comparative modeling for ABC transporters like P-gp, with initial models later refined using structures of mouse P-gp, which shares significant sequence identity with human P-gp [141,142,143,144]. These models provided insights into the transport mechanisms, potential substrate/drug-binding sites, and lateral portals for substrate entry [16,145,146,147,148,149].

**Figure 1:**
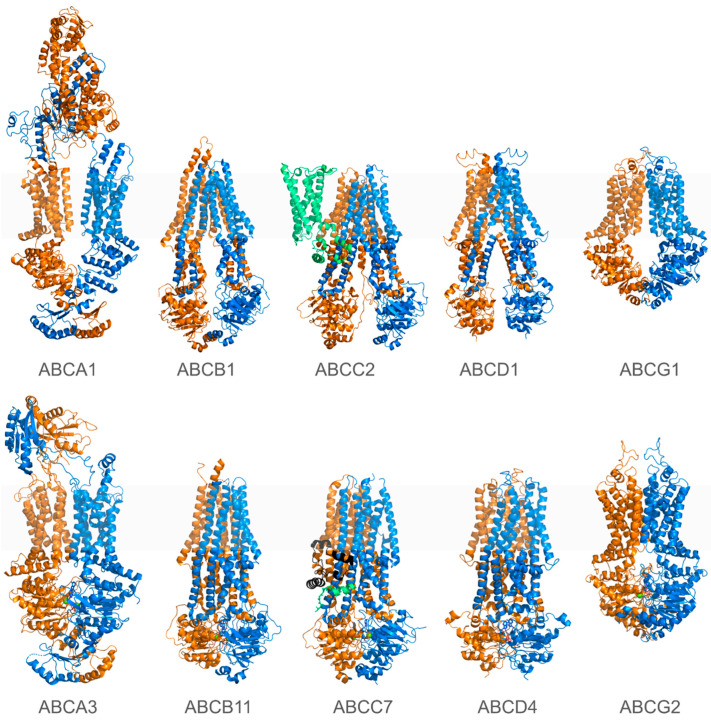
3D structures of human ABC transporters in inward-facing (**top**) and outward-facing (**bottom**) conformations. One representative member from the A, B, C, D, and G subfamilies is shown for each conformation. For the inward-facing state (top), the depicted transporters are ABCA1 (PDB: 7TDT), P-gp/ABCB1 (PDB: 6FN1), MRP2/ABCC2 (PDB: 8JY5), ABCD1 (PDB: 7VZB), and ABCG1 (PDB: 7R8D). For the outward-facing state (bottom), the transporters are ABCA3 (PDB: 7W02), BSEP/ABCB11 (PDB: 8PMD), CFTR/ABCC7 (PDB: 6O2P), ABCD4 (PDB: 6JBJ), and BCRP/ABCG2 (PDB: 6HBU). Transmembrane domain 1 (TMD1) and transmembrane domain 2 (TMD2) are represented in orange and blue, respectively. TMD0, specific to the C subfamily, is shown in green. For CFTR, the N-terminal lasso and the R domain are shown in black and green, respectively.

**Figure 2:**
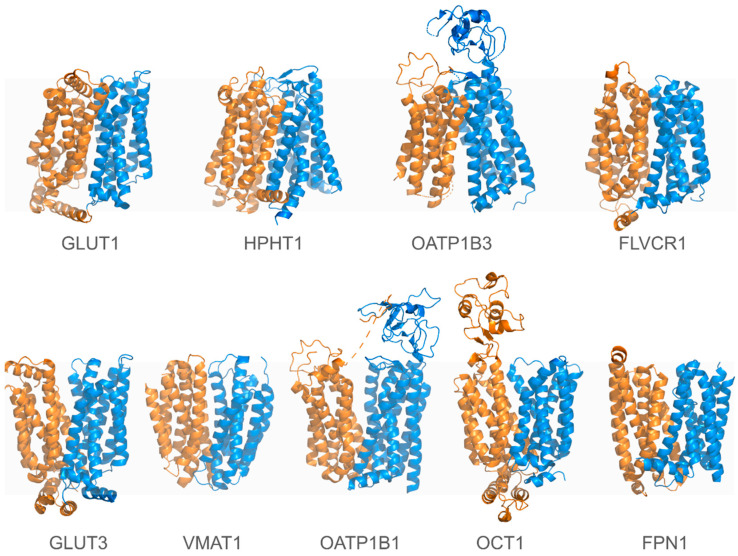
3D structures of human SLC transporters in inward-facing (**top**) and outward-facing (**bottom**) conformations. The inward-facing (IF) transporters depicted at the top are GLUT1/SLC2A1 (PDB: 8THA), HPHT1/SLC15A4 (PDB: 8WX5), OATP1B3/SLCO1B3 (PDB: 8PG0), and FLVCR1/SLC49A1 (PDB: 8UBW). The outward-facing (OF) transporters depicted at the bottom are GLUT3/SLC2A3 (PDB: 4ZWC), VMAT1/SLC18A1 (PDB: 8TGG), OATP1B1/SLCO1B1 (PDB: 8HNB), OCT1/SLC22A1 (PDB: 8ET8), and FPN1/SLC40A1 (PDB: 6W4S). The N-lobe and C-lobe are shown in orange and blue, respectively.

**Figure 3:**
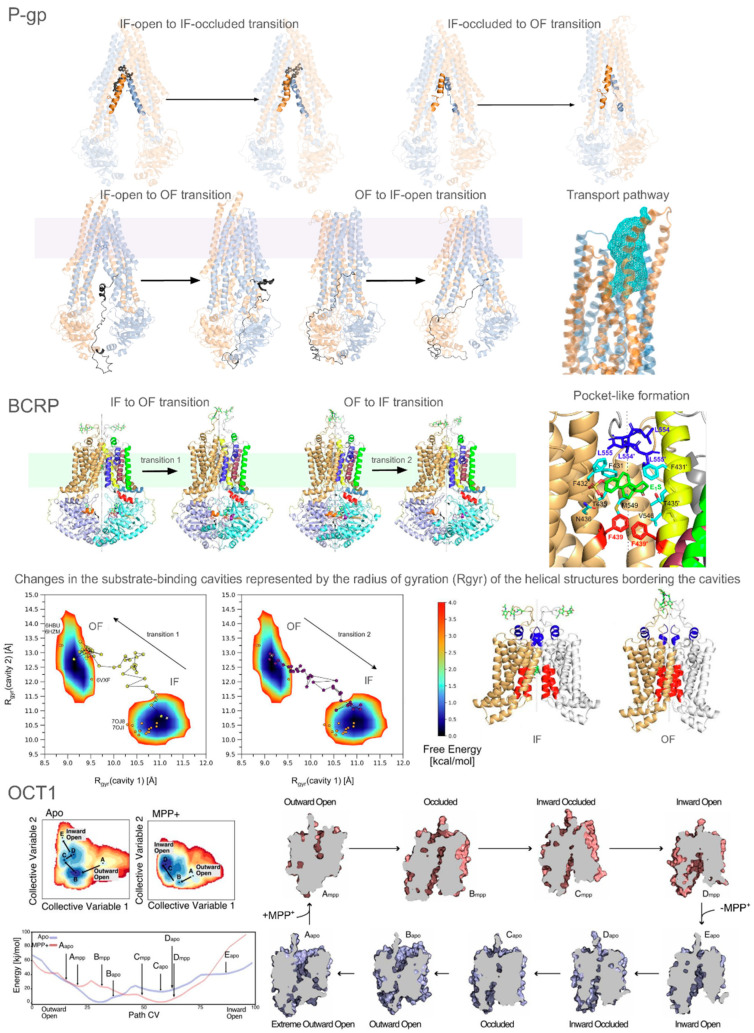
Proposed functional cycle and transport mechanisms of ABC and SLC transporters based on molecular modeling studies. ABCB1/P-gp simulations using ketMD and ABMD to depict transitions between IF-open, IF-occluded, and OF states in presence of ATP molecules. At the top, the transition from the IF-open to IF-occluded state is shown on the left in the presence of a P-gp inhibitor (encequidar), while the transition from the IF-occluded to OF state in absence of ligand is shown on the right. At the bottom (left/center), transitions between the IF-open and OF states are shown in the presence a P-gp substrate (vincristine) and in the presence of the linker region. The whole protein is shown in transparent, except for the linker domain (black, bottom left/center). Visualization of VIN occupancy density (bottom right) shows substrate translocation pathways through P-gp as proposed by Elbahnsi et al., CSBJ 2024 [156]. Adapted from Elbahnsi, A., Dudas, B., Cisternino, S., Declèves, X., & Miteva, M.A. “Mechanistic insights into P-glycoprotein ligand transport and inhibition revealed by enhanced molecular dynamics simulations” Computational and Structural Biotechnology Journal (2024), Volume 23, Pages 2548–2564. DOI: https://doi.org/10.1016/j.csbj.2024.06.010, licensed under Creative Commons BY-NC-ND 4.0. ABCG2/BCRP conformational transitions from inward-facing (IF) to outward-facing (OF), and vice versa, during ketMD simulations are shown (top left/center) as proposed by Dudas et al., CSBJ 2022 [157]. Additionally, a pocket-like formation (top right) is observed during substrate translocation, located between the F439 valve (in red) and the leucine gate (in blue), with key interacting residues labeled and shown in cyan licorice representation. Changes in the substrate-binding cavities represented by the radius of gyration (Rgyr) of the helical structures bordering the cavities. Adapted from Dudas, B., Decleves, X., Cisternino, S., Perahia, D., & Miteva, M.A. “ABCG2/BCRP transport mechanism revealed through kinetically excited targeted molecular dynamics simulations” Computational and Structural Biotechnology Journal (2022), Volume 20, Pages 4195–4205. DOI: https://doi.org/10.1016/j.csbj.2022.07.035, licensed under Creative Commons BY 4.0. SLC22A1/OCT1 thermodynamic conformational ensemble with and without substrate is depicted as proposed by Yee et al., Mol. Cell 2024 [160]. Left top panels show 2D conformational landscapes of apo and MPP^+^-bound OCT1, derived from enhanced-sampling MD simulations (AWH and steered MD). Left bottom panel presents a 1D projection of the free energy landscapes, comparing apo (blue) and MPP^+^ (red) along the outward-open to inward-open path. Right panels illustrates volume-filling models from low-energy states. The top panel shows the models in the presence of MPP^+^, while the bottom panel represents the apo form (without MPP^+^). Adapted from Molecular Cell, Vol 84, Issue 10, Sook Wah Yee et al., ‘The full spectrum of SLC22 OCT1 mutations illuminates the bridge between drug transporter biophysics and pharmacogenomics’, Pages 1932–1947.e10, Copyright 2024, with permission from Elsevier. DOI: https://doi.org/10.1016/j.molcel.2024.04.008.

Recent advancements in cryo-EM have significantly enhanced our understanding of ABC and SLC transporter structures, resolving them at near-atomic levels under various conditions (Figure 1 and Figure 2) [1,8]. These high-resolution 3D structures have elucidated the mechanisms of membrane transport and substrate specificity, emphasizing the importance of large conformational changes during substrate transport. Complementing these structural studies, advanced molecular modeling approaches, including deep learning algorithms like AlphaFold [150,151,152] and other advanced artificial intelligence (AI)-driven tools [153], have allowed the obtainment of highly relevant models for transporters lacking experimental structures. Docking, molecular dynamic (MD) simulations, and enhanced sampling techniques, such as metadynamics and steered MD [17,154,155,156,157,158,159,160,161], have further enriched our understanding by uncovering ligand–protein interactions and capturing the dynamic behavior and rare events associated with transporter function (Figure 3).

### 3.1. Structures, Functions, and Transport Mechanisms of ABC Transporters

Human ABC proteins are divided into seven subfamilies, with A, B, C, D, and G playing key roles in membrane transport. The E and F subfamilies, which lack transmembrane domains and are involved in cellular functions such as ribosome modulation, are not covered here. Table 1 lists human ABC transporters from subfamilies A, B, C, D, and G, for which experimentally determined structures are available.

ABC transporters share a common structural framework. They are composed of two transmembrane domains (TMDs), each consisting of six transmembrane (TM) helices that form the substrate-binding sites. These transmembrane helices are connected by extracellular loops (ECLs) on the outer side and intracellular regions, referred to as either intracellular helices (ICHs) or intracellular loops (ICLs), on the inner side of the plasma membrane. Some members of the C subfamily possess an additional TMD (Figure 1). ABC transporters also feature two nucleotide-binding domains (NBD1 and NBD2), arranged in a head-to-tail orientation, creating two nucleotide-binding sites (NBSs) at their interface. These sites are essential for ATP binding and hydrolysis, as well as for coordinating Mg^2+^ ions, thereby providing the energy necessary for their transport function. However, in some ABC transporters, such as CFTR, one of these sites is degenerated, resulting in reduced or absent ATP hydrolysis activity, which alters the transport mechanism. Key motifs within the NBS, such as the highly conserved ABC signature motif (LSGGQ), the Walker-A and Walker-B motifs, and the Q- and H-loops, are consistent features in the ABC transporter superfamily. In the ABCB and C subfamilies, a notable structural feature is the flexible, unstructured linker connecting the C-terminal end of NBD1 to the N-terminal part of TMD2. These linkers, often unresolved in experimental structures, play a critical role in regulating the activity and function of many ABC transporters through post-translational modifications [162,163,164].

**Table 1:** List of human ABC transporters with experimentally determined 3D structures available in the PDB, along with their known substrates, primary functions, and corresponding references.

Transporter	Substrates	Functions	References
ABCA1	phospholipids	phospholipid transfer to apolipoproteins	[165,166,167]
ABCA3	phospholipids	transport of phospholipids from the cytoplasm into the lumen side of lamellar bodies; participates in the lamellar bodies biogenesis and homeostasis of pulmonary surfactant	[168]
ABCA4	retinal-phosphatidylethanolamine conjugates	transport of retinal-phosphatidylethanolamine conjugates from the lumen to the cytoplasmic leaflet of photoreceptor outer segment disk membranes	[169,170,171]
ABCA7	phosphatidylserine	lipid homeostasis and macrophage-mediated phagocytosis	[172]
ABCB1	phosphatidylcholine, diverse compounds, xenobiotics, drugs, …	efflux pump responsible for decreased drug accumulation in multidrug-resistant cells	[173,174,175,176,177]
ABCB3	peptide antigens	mediates unidirectional translocation of peptide antigens from cytosol to endoplasmic reticulum	[178]
ABCB4	phosphatidylcholine	floppase translocating phosphatidylcholine from the inner to the outer leaflet of the canalicular membrane bilayer into the canaliculi of hepatocytes	[179,180]
ABCB6	porphyrins	importer of porphyrins from the cytoplasm into the mitochondria	[181,182,183,184]
ABCB7	glutathione-coordinated iron–sulfur cluster	allows assembly of the cytosolic iron-sulfur (Fe/S) cluster-containing proteins and participates in iron homeostasis	[185]
ABCB8	potassium	subunit of the mitochondrial ATP-gated potassium channel (mitoK (ATP))	[186]
ABCB10	mitochondrial biliverdin	export of substrate from the mitochondrial matrix to the cytosol	[187,188]
ABCB11	bile salts	transport of bile salts across the canalicular membrane of hepatocytes, hepatic bile acid homeostasis	[189,190,191]
ABCC2	conjugated organic anions, various substrates, drugs	active transport of various substrates including many drugs, toxicants and endogenous compound across cell membranes	[192]
ABCC3	bile acids, glucuronides, various drugs	transports various substrates including many drugs, toxicants, and endogenous compound across cell membrane, transports glucuronide conjugates and also various bile salts	[193]
ABCC4	cAMP and cGMP, bile acids, steroid conjugates, urate, prostaglandins, xenobiotics, drugs	extrudes physiological compounds and xenobiotics from cells, transports endogenous molecules that have a key role in cellular communication and signaling	[194,195]
ABCC7	chloride, bicarbonate	ion channel that plays an important role in the regulation of epithelial ion and water transport and fluid homeostasis	[68,196,197,198,199,200,201,202]
ABCC8	potassium	subunit of the beta-cell ATP-sensitive potassium channel (KATP), regulator of ATP-sensitive K^+^ channels and insulin release	[203,204]
ABCD1	(VLCFA)-CoA	transport of very-long-chain fatty acid (VLCFA)-CoA from the cytosol to the peroxisome lumen	[205,206,207,208]
ABCD4	cobalamin (vitamin B12)	transports cobalamin (vitamin B12) from the lysosomal lumen to the cytosol	[209]
ABCG1	phospholipids, cholesterol	efflux of phospholipids, active component of the macrophage lipid export complex	[210,211,212]
AGBCG2	diverse compounds, xenobiotics, drugs, …	extrudes a wide variety of physiological compounds, dietary toxins, and xenobiotics from cells	[213,214,215,216,217,218,219,220]
ABCG5-G8	cholesterol	obligates heterodimer mediating sterol transport across cell membrane, selective transport of sterols/cholesterol in and out of the enterocytes and in selective sterol excretion by the liver into bile	[212,221,222,223]

ABC transporters are dynamic proteins that cycle through multiple conformational states during substrate transport, specifically the inward-facing (IF) and outward-facing (OF), open and occluded states [19]. In the absence of ligands or nucleotides, ABC transporters typically adopt the IF conformation, characterized by separated NBDs and an inverted V-shaped opening toward the cell’s intracellular space, ready to bind substrates. Binding of substrates, inhibitors, or modulators triggers substantial conformational changes in the TMDs, which subsequently bring the NBDs closer together, transitioning the protein toward an occluded IF state. This state is marked by the formation of a closed central cavity that isolates the bound ligand from both intracellular and extracellular environments. While substrates and competitive modulators often bind at similar sites within this cavity, they can have different effects on the NBDs’ configuration and interaction, thereby influencing the transport process in distinct ways. Following substrate binding, ATP binding drives the transporter toward the OF conformation. In this state, the NBDs dimerize and the TMDs shift to present either an outward-facing V-shaped opening or a closed pathway, indicative of a post-substrate release state. ATP hydrolysis then resets the transporter back to the IF open state, ready for a new cycle. This functional process follows the alternating access model, which is broadly applicable across different ABC transporter subfamilies and is crucial for understanding their roles in cellular homeostasis and pathologies such as cancer drug resistance [19].

ABCB1/P-gp structural studies have provided high-resolution structures in multiple conformational states, including the initial inward-facing open (IF-open) conformation obtained from mouse models [127,128,129,130], and more recently, the inward-facing occluded (IF-occluded) state [173,174,175,176], resolved in the presence of various substrates and inhibitors, and the OF state in human P-gp [177]. In the IF-open state, P-gp displays a large central cavity accessible from both the plasma membrane and the cytoplasm capable of accommodating diverse substrates and inhibitors [141,224]. The IF-occluded state, characterized by kinked TM4 and TM10 helices, traps substrates within the binding pockets and may represent either an intermediate conformation or an inhibited state, particularly when stabilized by specific inhibitors. In this conformation, three distinct binding regions have been proposed by the Locher group [175], comprising a central drug-binding pocket, an access tunnel accessible starting at the TM4/TM10 gate, and a vestibule where the access tunnel connects with the central drug-binding pocket. The OF state, facilitated by ATP binding and NBD dimerization, reorients the binding pocket towards the extracellular side, enabling substrate expulsion [177].

MD simulations have been instrumental in further exploring P-gp conformational dynamics and its interactions with ligands. Studies have shown that substrates and inhibitors influence the conformational distribution of the protein differently [146,225]. Specifically, substrate binding brings the NBDs closer together, aligning them in a way that promotes ATP hydrolysis. In contrast, inhibitors stabilize the NBDs in a more separated configuration, which may hinder ATP hydrolysis and subsequently impede substrate transport. Targeted MD simulations have also been used to simulate the transitions between IF and OF states, revealing both translational and rotational movements of the NBDs during these conformational changes [226,227]. Additionally, steered MD and umbrella sampling have detailed interaction pathways and energy barriers associated with drug transport [154], while coarse-grained MD has provided insights into the role of the lipid environment in modulating P-gp function [228]. In our recent study employing adiabatic biased MD simulations, we investigated the unkinking mechanisms of the TM4 and TM10 helices during transitions from the occluded state (Figure 3). Our findings indicated that the initial unkinking of these helices is essential for the progression to the OF state, enabling subsequent NBD dimerization. This suggests that the kinked conformation serves as a regulatory checkpoint, preventing premature ATP hydrolysis and substrate transport until the helices are properly aligned. We should note that the IF-occluded state has been observed in the presence of an inhibitory antibody fragment, either with inhibitors or substrates. We proposed that the kinking and unkinking dynamics play a pivotal role in mediating communication between the TMDs and NBD2, representing a potential control for modulating P-gp activity [156].

The flexible linker region connecting NBD1 and TMD2, unresolved in current experimental structures due to its intrinsically disordered character, plays a significant role in regulating P-gp transitions through post-translational modifications [229,230,231]. Studies have investigated its positioning using modeling and MD [145,232]. Our simulations have suggested that this linker interacts with the intracellular helices, preventing the protein from achieving the OF conformation, thereby acting as a regulatory element (Figure 3). Its presence also facilitates access to the central cavities, indicating its involvement in P-gp function by influencing substrate binding and transport. These observations highlight the linker’s dual role in modulating both conformational transitions and ligand interactions [156]. Despite its critical role, the lack of high-resolution structural data for this region presents challenges, emphasizing the need for advanced techniques to elucidate its dynamics and interactions.

Early molecular docking studies, based on refined homology models derived from mouse structures, predicted several potential binding sites [233]. Ecker and colleagues developed an experimental data-guided docking strategy that exhaustively samples and clusters ligand poses to generate binding hypotheses for transporters like P-gp. This method involves analyzing structure–activity relationships (SARs) and validating these models through site-directed mutagenesis and structure-based pharmacophore models, which have proven useful for discovering novel inhibitors [234]. However, the inherent uncertainty in docking poses, especially for such flexible and promiscuous transport proteins, necessitates further refinement and validation of structure-based modeling. Ferreira and coworkers employed advanced molecular docking techniques and MD simulations using a refined murine P-gp structure to identify distinct binding sites, such as the M site (modulator), H site (Hoechst 33342), and R site (rhodamine 123) [233]. Their classification model successfully distinguished substrates from modulators, demonstrating significant predictive accuracy and emphasizing the importance of incorporating MD simulations for a comprehensive understanding of ligand–transporter interactions. Recent studies have also uncovered new binding sites outside the TMD main binding cavity, targeted by non-competitive inhibitors such as flavonoids [148,235]. These inhibitors bind at the interface between the TMDs and NBD2, suggesting that they may inhibit P-gp by limiting the conformational flexibility necessary for substrate translocation. This discovery offers a promising new strategy for overcoming multidrug resistance. These insights underscore the complexity of P-gp’s conformational landscape and highlight the need for advanced modeling techniques to gain a deeper understanding of its functional mechanisms.

Structural details of ABCG2/BCRP were elucidated through cryo-EM studies, capturing multiple conformational states of this homodimeric transporter in both OF and IF states under turnover conditions and in the presence of substrates and inhibitors [213]. Unlike other ABC transporters such as P-gp, which have a TMD–NBD arrangement in both halves, BCRP is a “half-transporter” with a distinctive NBD–TMD configuration, where the NBD precedes the TMD in each monomer, contributing to its unique transport mechanism [213]. Although higher-order oligomeric forms such as tetramers and dodecamers have been observed, BCRP must form a homodimer to be fully functional. It is thus suggested that these higher oligomeric forms are predominant in plasma membranes and may play a regulatory role, modulating the activity and stability of the functional dimeric BCRP [236]. The high-resolution structures have revealed the intricate architecture of BCRP, with its NBDs responsible for ATP binding and hydrolysis and its TMDs containing at least two distinct substrate-binding sites. These sites are separated by a leucine gate that regulates substrate access and transport across the membrane [213].

Modeling, docking, and MD simulation studies have been crucial in bridging gaps left by experimental methods, providing detailed structural insights into BCRP [237,238,239,240,241,242]. These studies have elucidated key interactions between inhibitors and BCRP, confirming the stability of various compounds within the substrate-binding cavity. Enhanced MD techniques, such as metadynamics, have further elucidated the substrate translocation pathways and the role of key residues in modulating the transporter’s activity [155]. Notably, we developed an innovative approach to enhance molecular dynamic simulations, kinetically excited targeted MD (ketMD), and successfully simulated the transitions between IF and OF states in both directions, as well as the transport of the endogenous substrate estrone 3-sulfate. We discovered an additional transient pocket between the two substrate-binding cavities and found that the presence of the substrate in the first cavity is essential to couple the movements between the TMDs and NBDs (Figure 3). This coupling is disrupted in the absence of a substrate, leading to an uncoupled state where the transporter is unable to complete the transport cycle. These findings underscored the critical role of substrate presence in maintaining the functional dynamics of BCRP [23].

Despite the progress made through cryo-EM and MD simulations, several challenges remain. The dynamic nature of BCRP and its ability to accommodate a wide variety of substrates complicate the prediction of drug-binding affinities and transport behavior. The lack of high-resolution structures for intermediate states during the transport cycle and for various substrates in some cavities limits our understanding of the complete transport mechanism. Additionally, the interplay between lipid molecules and the transporter, as observed in MD simulations, adds another layer of complexity. Understanding how the lipid environment influences BCRP’s conformational dynamics and substrate specificity is essential for developing effective inhibitors. Further advancements in enhanced sampling techniques and integrative modeling approaches are needed to fully characterize the dynamic landscape of BCRP and to design inhibitors that can effectively overcome MDR.

In recent years, a major breakthrough in cystic fibrosis research was made with the publication of experimental 3D structures of full-length CFTR proteins, initially from zebrafish [243,244] and subsequently from humans [68,196,197,198,199,200,201,202], solved using cryo-EM. The first zebrafish structure captured CFTR in an IF conformation representing a non-phosphorylated, ATP-free, quiescent (apo) state. Despite limited electron density for secondary structures, part of the unique regulatory (R) region of CFTR was observed between the two NBDs, preventing their dimerization and subsequent channel opening [243]. Then, a phosphorylated, ATP-bound conformation of zebrafish CFTR was reported, showing the R region shifted from its inhibitory position and the NBDs forming a head-to-tail dimer. However, the channel remained closed at the extracellular surface, indicating that the experimental conditions did not allow channel opening, despite phosphorylation and ATP binding [244]. These cryo-EM structures have also revealed a unique “lasso” conformation of the N-terminal region, which inserts into the membrane and packs against the transmembrane helices, as well as a notable discontinuity at helix TM8, accompanied by a displacement of TM7, differentiating CFTR from other ABC transporters [243,244]. Subsequent human CFTR structures confirmed these features [196,197] and further included the F508del mutant, providing crucial insights into how this mutation disrupts CFTR function [68]. Several CFTR structures have been solved in the presence of correctors, potentiators, and inhibitors, providing a valuable foundation for understanding the mechanisms underlying CFTR modulation [68,198,200,202,245]. Of note, the 3D structures of thermally stabilized chicken CFTR, despite being solved at low resolution, revealed a unique and different repositioning of the transmembrane helices between its inactive and active states [246].

Prior to the availability of high-resolution cryo-EM structures, modeling studies, including homology modeling and MD simulations, played an important role in advancing our understanding of CFTR’s functional architecture and mechanisms. Callebaut and coworkers significantly contributed to the field by identifying unique features of CFTR, such as the presence of specific salt bridges in the conductive state of the channel and lateral portals at the intracellular loops (ICLs) within the transmembrane helices, which facilitate ion flow from the cytosol toward the central pore [247]. These first structural insights, supported by experimental data [248,249,250,251], have been crucial for understanding the intricate gating mechanism of CFTR and its functional architecture. Notably, they applied metadynamics to explore the transitions between distinct states of CFTR, revealing the energy barriers associated with these transitions and identifying the conformational shifts that underlie channel gating [252].

**Figure 4:**
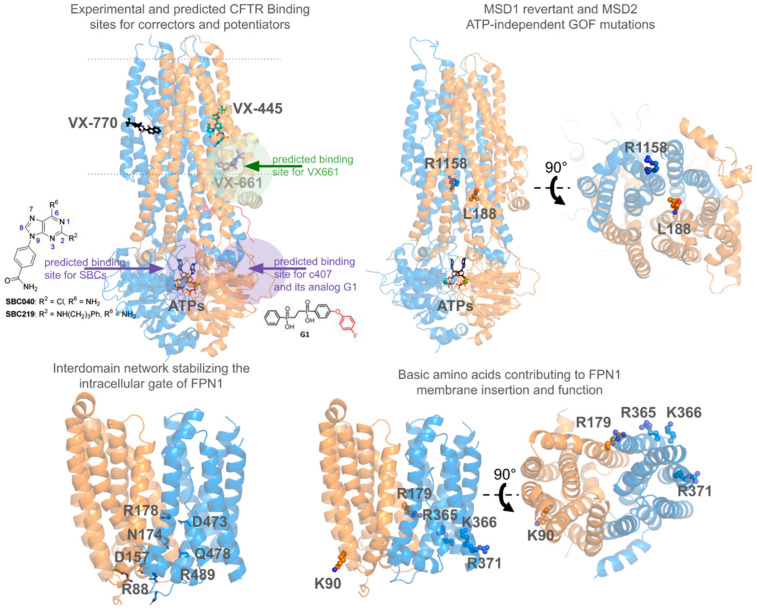
CFTR and FPN1 modeling and experimental insights from collaborative studies by Callebaut, Hinzpeter, and Le Gac groups and their coworkers. For CFTR (top panels), the left panel shows the binding sites of modulators, with correctors VX-661 in blue and VX-445 in cyan, [68,202] and the potentiator VX-770 in black [198] represented in stick and spheres. These molecules were extracted from cryo-EM structures referenced above. Notably, type I correctors VX-809 and VX-661 bind to the same site, which was previously predicted before being experimentally observed and highlighted here by the green-filled circle [253]. The C407 corrector and SBC potentiators binding sites within the cytoplasmic domains are shown by violet-filled circles [256,257]. On the right, TMD1 mutation L118W reverts class II mutatio L206W [254], while mutation R1158T in TMD2 enables ATP-independent gain of function for WT and gating mutants (e.g., G551D) [255]. For FPN1 (bottom panels), the left panel highlights the identification of an interlobe network of salt bridges and H-bonds involving key amino acids stabilizing the intracellular gate within the outward-facing (OF) conformation [261]. The right panel shows four basic amino acids interacting with the membrane bilayer, in proximity to residues from the intracellular gate. Experimental and computational studies suggest their importance in membrane insertion, regulation of the intracellular gate, and overall functions of FPN1 [262].

Additionally, the groups of Callebaut, Hinzpeter, and their collaborators combined mutagenesis experiments, functional assays, docking, and MD simulations to predict the impact of specific mutations on CFTR stability and function. They identified how pharmacological correctors like lumacaftor/VX-809 and tezacaftor/VX-661 interact with specific sites on the CFTR protein, stabilizing its structure and enhancing its function. Simulations demonstrated that these correctors improve intra-domain folding and inter-domain assembly by binding to distinct regions on CFTR, effectively rescuing the misfolded F508del mutation (Figure 4) [253]. Notably, one of these binding sites in TMD1 was later confirmed by cryo-EM studies, providing experimental validation and offering new insights into the mechanistic action of these drugs [202]. These foundational findings set the stage for interpreting cryo-EM structures and guided the development of targeted therapies for cystic fibrosis.

Their combined computational structural studies, along with mutagenesis and functional assays, also enabled a detailed investigation of the impact of various CFTR mutations, identifying key revertant/suppressor mutants that can partially correct the misfolded structure of disease-causing mutations (Figure 4) [254]. These mutants provided valuable insights into the structural requirements for CFTR stability and function, particularly emphasizing the importance of the TMD1 domain in proper folding, inter-domain communication, and channel gating. Moreover, recent findings showed that certain mutations within the TMD2 domain increase channel activity and enable CFTR opening by potentiators like ivacaftor/VX-770 without the need for cAMP elevation, suggesting an ATP-independent mechanism of action (Figure 4). These mutations showed additive effects with other gain-of-function mutants and affected the sensitivity and response to potentiators, highlighting the critical role of the TMD2 region in regulating CFTR activity and its potential as a therapeutic target for cystic fibrosis [255].

In collaboration with others, they identified binding sites for new compounds on CFTR, which demonstrated a synergistic effect when combined with existing therapies (Figure 4) [256,257]. Compound optimization, guided by docking and MD simulations, showed that targeting these additional sites can significantly enhance the efficacy of CFTR correctors. This strategy highlights the potential of combining multiple compounds to achieve greater therapeutic benefits for cystic fibrosis.

Other groups have utilized advanced computational methods to explore CFTR function and its pharmacological modulation. For instance, one study employed comparative modeling to investigate the thermodynamic interactions within the multi-domain structures of CFTR, particularly focusing on how the F508del mutation responds to lumacaftor/VX-809 [258]. Well-tempered metadynamic simulations were carried out to explore the dynamics of chloride within the channel, uncovering intricate insights about chloride pathways and binding sites [259]. Additionally, MD simulations under a strong hyperpolarizing electric field facilitated chloride passage through the bottleneck region of the channel, suggesting that such an artificial setup could induce the protein to adopt a functionally open state [260].

Despite these advances, several challenges remain. The fully open state of CFTR, which is essential for understanding the complete gating mechanism, has not been captured yet by experimental or modeling studies. Additionally, the structure, dynamics, and role of the regulatory (R) region are not fully elucidated, leaving a gap in our understanding of how this domain modulates CFTR activity. Moreover, certain CFTR mutants are not fully rescued by current therapies [73], underscoring the need for continued research to develop more effective strategies for correcting dysfunctional CFTR and to explore new avenues for drug discovery and design.

### 3.2. Structures, Functions, and Transport Mechanisms of SLC Transporters

The SLC transporters represent the largest group of transport proteins, comprising around 455 members clustered into 66 distinct families [14,90]. Recent advances in cryo-EM have provided valuable structural insights for over 60 different human SLC transporters in the PDB. However, for many SLCs, only one or two conformations have been characterized, leaving much of their transport cycle unexplored. Table 2 lists some human SLC transporters for which experimentally determined 3D structures are available. New 3D structure prediction algorithms like AlphaFold [150], combined with computational methods such as docking and MD simulations, hold great potential for advancing our understanding of the structural basis for SLC function [4,17].

Unlike ABC transporters, SLCs exhibit remarkable structural diversity, encompassing various unrelated folds. Notably, the major facilitator superfamily (MFS) and LeuT folds are common in human SLCs. Despite this diversity, SLC transporters share primary transport mechanisms—facilitated diffusion or secondary active transport—distinguishing them from ATP-driven transporters. Moreover, shared sequence motifs enable subfamily categorization and key functional elements are conserved among distinct subfamilies sharing the same structural class. For instance, the MFS structural core consists of twelve TM helices organized into two similar lobes: the N-lobe (TM1–TM6) and the C-lobe (TM7–TM12) (Figure 2) [14,15,263].

SLC transporters function through an alternating access mechanism, transitioning between key conformations: outward-open, outward-occluded, occluded, inward-occluded, and inward-open. (The occluded conformation prevents simultaneous access to the substrate-binding site from either side of the membrane). SLCs generally act as facilitative uniporters driven by concentration gradients or as secondary active transporters. In the latter, they couple the movement of one molecule against its gradient with the transport of another molecule down its gradient, either in a symport (same direction) or antiport (opposite direction) manner [95]. A recent cryo-EM structure of the sea urchin Na^+^/H^+^ exchanger (SLC9C1) illustrates the structural diversity of secondary active SLC transporters. This unique transporter combines a classical solute carrier unit with regulatory domains typically found in ion channels, such as a voltage sensor domain (VSD) and a cyclic nucleotide-binding domain (CNBD). These features enable a secondary-active transport mechanism that is regulated by changes in membrane voltage, showcasing a novel chimera mechanism [91].

Three primary transport models are proposed for SLCs: the “rocker-switch”, “gated-pore”, and “elevator” models [264].

The rocker-switch mechanism is characterized by conformational changes between outward-open and inward-open states. Initially, the transporter binds a substrate in the outward-open state, triggering a conformational shift to the inward-open state, allowing substrate release to the intracellular environment. Following substrate release, the transporter reverts to the outward-open state, ready for another cycle. This process, driven by various gradients (e.g., ion or substrate concentration gradients), is typical of the MFS fold. Understanding this mechanism is crucial for deciphering SLC roles in nutrient uptake, ion transport, and drug transport.

The gated-pore mechanism involves distinct mobile and static domains within the transporter. Upon substrate binding, the mobile domain undergoes a conformational change, effectively “opening” a gate to allow substrate passage through the membrane. After the substrate release, the mobile domain returns to its original position, “closing” the gate. The LeuT fold, seen in neurotransmitter transporters, exemplifies this mechanism, which is critical for the specific transport of a wide range of molecules involved in cellular homeostasis and signaling.

In the elevator mechanism, a mobile domain containing the substrate-binding site moves vertically along a stationary scaffold domain, akin to an elevator’s movement. When a substrate binds, the mobile domain shifts across the membrane, transporting the substrate to the opposite side. This mechanism is efficient for transporting substrates against their concentration gradients and is integral to various transporters involved in essential biological processes.

Recent cryo-EM studies have elucidated the structural basis of OCTs (SLC22 family), revealing how they mediate the uptake of organic cations like metformin through the alternating access model. Key residues and conserved motifs have been identified as orchestrators of state transitions during the transport cycle [265,266,267,268]. Inhibition studies have demonstrated how molecules like spironolactone can lock the transporter in specific conformations, blocking substrate translocation and providing a mechanistic basis for developing OCT inhibitors [266,268]. Integrative approaches combining deep mutational scanning, coevolutionary analysis, and advanced MD simulations have expanded our understanding of OCT1’s dynamic behavior, linking genetic variants to pharmacogenomic outcomes and drug responses (Figure 3) [160]. These insights lay a foundation for precision medicine and therapeutic development targeting OCTs.

The rat OAT1 structure, resolved by cryo-EM, provided the first insights into substrate and inhibitor binding mechanisms [269,270]. However, the lack of high-resolution human OAT structures remains a challenge. Computational models of human OAT1, supported by MD simulations, have revealed key features, such as (i) a conserved “charge-relay system” involving key residues acting as molecular switches that mediate conformational changes, and (ii) the crucial role of lipid interactions (particularly with phosphatidylethanolamine and cholesterol) in maintaining stability and function [271,272]. Future efforts to resolve human OAT structures and capture transient states will be crucial for developing specific inhibitors with reduced off-target effects.

Similarly, structural studies of FPN1/SLC40A1 have greatly enhanced our understanding, first through the characterization of its bacterial homologue, BbFpn, by Taniguchi et al. using X-ray crystallography, confirming the typical MFS fold and offering key insights into the iron transport mechanism and regulation by hepcidin [273]. These structures revealed a unique split in TM7, essential for conformational flexibility and gating during transport. Deshpande et al. later suggested that FPN1 relies on extracellular Ca^2^^+^ for effective iron transport, as shown in the Ca^2^^+^-bound BbFpn IF structure [274]. In this scenario, the “alternating access” mechanism involves Ca^2^^+^ binding in the OF state, which induces a conformational change to the IF state, allowing Fe^2^^+^ to bind. This then triggers a return to the OF state, where Fe^2^^+^ is released and oxidized to Fe^3^^+^ for systemic distribution via transferrin.

More recent cryo-EM studies have further advanced our understanding of human FPN1 in various functional states. Billesbølle et al. first solved the OF structure of HsFPN1 in lipid nanodiscs, both in the apo state and bound to hepcidin and cobalt. Their findings clarified the two iron-binding sites, in the N- and C-lobes, suggesting a potential coupling between these sites and hepcidin binding, influenced by the mobility of TM7b [275]. Subsequently, Pan et al. used cryo-EM to resolve the OF structures of FPN1 from the Philippine tarsier (TsFpn) in the presence and absence of hepcidin. They confirmed two iron-binding sites and demonstrated that Fe^2^^+^ export is coupled with proton (H^+^) transport, supporting the hypothesis that FPN1 may function as an Fe^2^^+^/H^+^ antiporter [276].

Adding to this body of knowledge, Lehmann et al. described the first cryo-EM structures of human FPN1 in complex with the clinical-stage inhibitor vamifeport. Their study revealed the transporter in both OF and OF-occluded conformations, shedding light on the intricate gating mechanism. The OF-occluded state is particularly intriguing, as it provides a snapshot of FPN1 during its conformational transition, with the extracellular entrance sealed off by rearrangements in the TM7b helix. This conformational state is stabilized by vamifeport, which binds at the N- and C-domain interface, disrupting the conformational changes required for iron transport and offering a structural basis for its competitive inhibition of FPN1 against hepcidin [137].

The collaborative effort between the Callebaut and Le Gac groups has provided crucial additional insights into the structural and functional characteristics of FPN1. Through a combination of molecular modeling and dynamics, mutational analysis, and biochemical assays, they have elucidated the role of critical residues in iron transport, the impact of disease mutations, and the influence of lipid interactions on FPN1 stability and activity (Figure 4). Notably, their work has shed light on the intricate network of interactions that govern the intracellular gate, revealing how disruptions in this network can impair iron export and contribute to ferroportin-related diseases [134,261,262,277]. These findings have significantly advanced the understanding of FPN1’s mechanistic function in health and disease.

**Table 2:** List of some human SLC transporters with experimentally determined 3D structures available in the Protein Data Bank (PDB), along with their known substrates, primary functions, and corresponding references.

Transporter	Substrates	Functions	References
GLUT1/SLC2A1	glucose	facilitative glucose transporter, which is responsible for constitutive or basal glucose uptake	[278,279,280]
GLUT3/SLC2A3	glucose	mediates the uptake of glucose, 2-deoxyglucose, galactose, mannose, xylose and fructose	[281,282]
GLUT4/SLC2A4	glucose	insulin-regulated facilitative glucose transporter, which plays a key role in removal of glucose from circulation	[283]
SGLT1/SLC5A1	glucose/Na^+^	electrogenic Na^+^-coupled sugar symporter that actively transports D-glucose or D-galactose at the plasma membrane, driven by a transmembrane Na^+^ electrochemical gradient set by the Na^+^/K^+^ pump	[284]
SGLT2/SLC5A2	glucose/Na^+^	electrogenic Na^+^-coupled sugar symporter that actively transports D-glucose at the plasma membrane, driven by a transmembrane Na^+^ electrochemical gradient set by the Na^+^/K^+^ pump	[285,286,287]
HsPepT1/SLC15A1	oligopeptides	electrogenic proton-coupled amino-acid transporter that transports oligopeptides, primarily responsible for the absorption of dietary di- and tripeptides from the small intestinal lumen	[110]
HsPepT2/SLC15A2	oligopeptides	electrogenic proton-coupled amino-acid transporter that transports oligopeptides	[110]
PHT2/SLC15A3	peptide histidine	proton-coupled amino-acid transporter that transports free histidine and certain di- and tripeptides	
HPHT1/SLC15A4	L-histidine GlySar dipeptide	proton-coupled amino-acid transporter that mediates the transmembrane transport of L-histidine and some di- and tripeptides from inside the lysosome to the cytosol, and plays a key role in innate immune response	[288]
MCT1/SLC16A1	monocarboxylate	transport across the plasma membrane of many monocarboxylates; contributes to the maintenance of intracellular pH	[289,290]
MCT2/SLC16A7	monocarboxylate	proton-coupled monocarboxylate symporter; transport across the plasma membrane of monocarboxylates	[291]
SIALIN/SLC17A5	nitrates	anion transporter that operates via 2 distinct transport mechanisms: proton-coupled anion cotransport and membrane potential-dependent anion transport; exports glucuronic acid and free sialic acid derived from sialoglycoconjugate degradation out of lysosomes	[292]
VAT1/VMAT1/SLC18A1	H^+^/monoamine	electrogenic antiporter that exchanges one cationic monoamine with two intravesicular protons across the membrane of secretory and synaptic vesicles; transports catecholamines and indolamines with higher affinity for serotonin	[293]
VAT2/VMAT2/SLC18A2	H^+^/monoamine	electrogenic antiporter that exchanges one cationic monoamine with two intravesicular protons across the membrane of secretory and synaptic vesicles; transports a variety of catecholamines such as dopamine, adrenaline and noradrenaline, histamine, and indolamines such as serotonin	[294,295,296]
OATP1B1/SLCO1B1/LST-1/OATP-C/SLC21A6	organic anion	mediates the uptake of organic anions; broad substrate specificity, can transport both organic anions and conjugated steroids	[297,298]
OATP1B3	organic anion	mediates the uptake of organic anions; broad substrate specificity, can transport both organic anions and conjugated steroids	[297]
OCT1/SLC22A1	organic cation	transport of a variety of organic cations such as endogenous bioactive amines, cationic drugs and xenobiotics; functions as a pH- and Na^+^-independent, bidirectional transporter	[265,266,267]
OCT2/SLC22A2	organic cation	transport of a variety of organic cations such as endogenous bioactive amines, cationic drugs and xenobiotics	[265]
OCT3/SLC22A3	organic cation	transport of a variety of organic cations such as endogenous bioactive amines, cationic drugs and xenobiotics; functions as a Na^+^- and Cl^−^-independent, bidirectional uniporter	[268]
SPNS2	sphingosine-1-phosphate	exports S1P via facilitated diffusion; required for the egress of T-cells from lymph nodes during an immune response by mediating S1P secretion	[299,300,301]
FLVCR1	heme	heme b transporter that mediates heme efflux from the cytoplasm to the extracellular compartment	[302,303]
FLVCR2	heme	putative heme b importer involved in heme homeostasis in response to the metabolic state of the cell	[303]
FPN1/SLC40A1	iron (Fe^2+^)	transports Fe^2+^ from the inside of a cell to the outside of the cell, playing a key role for maintaining systemic iron homeostasis	[137,275]

Key questions remain about FPN1’s mechanism for using the proton motive force to transport Fe^2^^+^, including whether it acts as a symporter or antiporter and the role of specific residues and lipids in this process. Evidence suggests overlapping but distinct iron and proton binding sites, indicating a complex interplay of conformational states. Stabilizing interactions, such as salt bridges and hydrogen bonds, are crucial for maintaining OF and IF conformations, with mutations often linked to iron overload disorders. Understanding these molecular details, including the role of lipids in MFS protein function, remains an important research focus.

## 4. Conclusions, Challenges, and Future Directions

ABC and SLC transporters are fundamental to numerous physiological processes, mediating the transport of a diverse array of substrates, endogenous compounds, and drugs across cellular membranes. While ABC transporters primarily use ATP hydrolysis to drive the efflux of substrates, SLC transporters exhibit remarkable structural and functional diversity, operating through various mechanisms, including facilitated diffusion and secondary active transport. Recent advances in structural biology, particularly cryo-EM, have significantly deepened our understanding of these complex proteins, revealing intricate details of their conformational states, substrate binding, and transport mechanisms. Computational approaches, including MD simulations, have further complemented experimental findings, offering dynamic perspectives on transporter behavior and elucidating mechanisms that remain elusive to traditional experimental techniques.

While molecular studies have significantly advanced our understanding of transporter functions, they also underscore the complexity of these proteins. Often large and polyspecific binding cavities of transporters make modeling their interactions with ligands particularly challenging, necessitating cautious interpretation and validation of computational results. The integration of homology modeling, conventional MD simulations, and enhanced sampling techniques elucidates structural and functional aspects of ABC and SLC transporters. However, ongoing challenges include improving the accuracy of model predictions, efficient sampling of conformational states, and incorporating experimental data to validate computational results. To gain a comprehensive understanding of protein dynamics and functions, it is essential to embrace a conformational continuum view of biomolecules. This involves capturing the entire spectrum of protein conformations, starting from a static structure obtained through methods like cryo-EM, X-ray crystallography, or modern modeling tools such as AlphaFold 3 [151] based on artificial intelligence (AI). MD simulations, while making significant progress in recent years, face two fundamental challenges, the first of which is describing the interactions among all particles using molecular mechanics force fields. Indeed, achieving a complete and precise description of all interactions at the quantum chemistry level is practically unfeasible. Second, a sampling problem persists due to the computational resources required for simulations, effectively limiting their timescale to the microsecond-to-millisecond range depending on the studied system size. For example, Lindorff-Larsen et al. used specialized supercomputers like Anton to simulate fast-folding proteins, with the largest system comprising approximately 80,000 atoms, over millisecond timescales, capturing folding and unfolding events [304]. However, for larger systems like ABC and SLC transporters, classical atomistic MD simulations often fall short in capturing transitions between IF and OF states due to the extended timescales and complex conformational changes involved. Consequently, enhanced sampling methods have been developed, employing various strategies to explore a broader conformational space than classical MD simulations (see enhanced sampling techniques reviewed in [305]). These methods enable the observation of rare events that can occur on millisecond-to-second timescales, even while the simulations are run over just a few nanoseconds to microseconds.

More recently, AI techniques like machine learning and deep learning (recently reviewed in [6]) have become central to analyzing large molecule datasets to elucidate transporter–ligand interactions for predicting putative substrates, inhibitors, and modulators, representing a significant advancement in the field. Moreover, more data have become available thanks to projects like RESOLUTE and EUbOPEN aiming to experimentally resolve the 3D structures of a large number of SLCs and finding new chemical probes for these transporters.

Despite these significant advances, our knowledge of ABC and SLC transporters is still incomplete. The complexity of these proteins, which are regulated by various intracellular and extracellular factors, poses substantial challenges for both experimental and computational studies. Of note, the intrinsically disordered and experimentally unresolved regions, such as the linker in P-gp, the R region in CFTR, and other large extracellular loops, are particularly challenging to model accurately, even with advanced computational techniques. Moreover, mutations in these transporters, linked to various diseases, significantly alter their function, highlighting the need for research focused on understanding these changes, especially in the context of genetic variability across populations, to better predict individual drug responses and design targeted therapies. Furthermore, the regulation of these transporters by post-translational modifications, protein–protein interactions, and lipid environments complicates predictions of their functional consequences. A critical area requiring further investigation is the interaction of these transporters with membrane lipids, which is essential for a deeper understanding of their function.

To overcome these challenges, integrating high-resolution structural data from cryo-EM and large-scale pharmacology data with computational methods such as advanced MD simulations and AI-driven modeling can bridge the gap between static structures, the dynamic picture, and biological phenomena at multiscale levels. That will ensure complete understanding of the roles of SLC and ABC transporters in drug pharmacokinetics and pharmacodynamics, their involvement in MDR and DDIs, and will accelerate the development of more effective therapeutics, overcoming drug resistance and advancing personalized medicine.
